# Mechanisms of possible self-limitation in the invasive Asian shore crab *Hemigrapsus sanguineus*

**DOI:** 10.1038/s41598-020-74053-5

**Published:** 2020-10-09

**Authors:** Blaine D. Griffen, James Bailey, Jade Carver, Ashley Vernier, Eleanor R. DiNuzzo, Lars Anderson, Morgan Meidell, Ben Potter

**Affiliations:** Biology Department, Brigham Young University, Provo, UT 84662 USA

**Keywords:** Ecophysiology, Invasive species

## Abstract

Population sizes of invasive species are commonly characterized by boom-bust dynamics, and self-limitation via resource depletion is posited as one factor leading to these boom-bust changes in population size. Yet, while this phenomenon is well-documented in plants, few studies have demonstrated that self-limitation is possible for invasive animal species, especially those that are mobile. Here we examined the invasive Asian shore crab *Hemigrapsus sanguineus*, a species that reached very high abundances throughout invaded regions of North America, but has recently declined in many of these same regions. We examined the relationship between diet, energy storage, reproduction, and growth in crabs collected from the New Hampshire coast. We show that energy storage and reproduction both increase with diet quality, while growth declines with diet quality. These results suggest that self-limitation may be a contributing factor to the recent declines of *H. sanguineus* at sites where this invader was once much more abundant. Further, these results suggest a diet-associated tradeoff in energy allocation to different vital rates, with a focus on reproduction when high quality resources are consumed, and a focus instead on growth when poor quality resources are consumed.

## Introduction

The concept that no population can increase forever and that something limits all populations from growing without bounds is a fundamental maxim of ecology^1^. Limits to population growth can occur via numerous mechanisms, including density-dependent predation^2^, interspecific and intraspecific competition for limiting resources^3,4^, and density-dependent spread of disease^5^. Self-limitation occurs via a subset of regulatory mechanisms where endogenous factors limit individual performance or survival, and these individual limitations in turn restrict population growth. The concept of self-limitation has been posited as one of the fundamental “laws” of population ecology^6^. Self-limitation can have important ecological consequences that extend beyond prohibiting runaway population growth, including facilitating the persistence of rare species in communities^7^ and shaping the evolution of life-history traits^8^.

Self-limitation can occur across a wide range of systems and through various processes, but often has resource limitation at its roots. Resource limitation can occur because of several factors, including territorial defense that limits the sharing of resources^9^, environmental variation in resource availability^10^, and competition for resources among conspecifics^11^. This resource limitation in turn alters vital rates so that under food-limited conditions, growth rates^12^, survival rates^13^, and reproductive rates^14^ all decline. These changes in vital rates may also interact. As increased food availability can lead to increased fecundity via more rapid growth rates, because larger body sizes in females can support the production of more eggs^15^. In addition to the amount of food, diet quality can also alter vital rates^16,17^. This can occur because of life history tradeoffs that set in when resources are limited^18^, where energy or nutrient shortfalls limit the ability of individual organisms to simultaneously achieve optimal performance in survival, growth, and reproduction. All these examples demonstrate mechanisms through which self-limitation via food availability or quality can limit the growth of a population.

Invasive species may often be regulated by self-limitation. Invasive populations commonly exhibit periods of rapid growth shortly after invasion, followed by periods of reduced growth and even population decline (i.e., a boom-bust pattern)^19,20^. Boom-bust patterns are often attributed to self-limitation due to lack of resources^19^. However, while self-limitation is often invoked to explain these patterns in invasive species, it is rarely demonstrated. Further, the mechanistic role of life history tradeoffs in this self-limitation, triggered by reduced quality or abundance of food intake, remains unknown. A literature search using the Web of Science (search terms: “self-limitation” AND “invasive species”; or the terms “resource depletion” AND “invasive species” AND “reproduction”; or the terms “density dependent” AND “invasive species” AND “reproduction”) yielded numerous studies in plant systems, but only three studies in animal systems that have demonstrated the possibility for self-limitation, and none of them demonstrated a mechanistic link between resource availability and population vital rates^21–23^.

We examined the Asian shore crab *Hemigrapsus sanguineus*, which is invasive to the east coast of North America^24^ and to Europe^25^*.* On the North American east coast, *H. sanguineus* was first noted in 1988 in Cape May, New Jersey^24^, where it was most likely introduced via ballast water, perhaps multiple times^26^. Over the next few years it rapidly spread from mid-coast Maine to North Carolina^26^, where *H. sanguineus* displaced other species of crabs^27,28^ and became the numerically dominant crab species over much of this range^29,30^. High densities of 100–200 individuals m^-2^ have been documented throughout much of its range^28,31–33^. However, after several years of maintaining these high population densities, *H. sanguineus* has recently declined in some areas to approximately half its former densities^33–36^.

Several factors may contribute to population declines, including predation, competition, disease or parasites, etc., and each of these ecological interactions impose limits to invasive crabs in general^37–41^. However, previous work suggests that these mechanisms may have relatively little impact on Asian shore crabs in their invaded range. For instance, existing evidence suggests that common potential predators do not heavily prey on Asian shore crabs^42–44^. Additionally, competition does not seem to play a major role, as this species has the upper hand in interspecific interactions with other intertidal crabs^28,40^ and displays fairly weak conspecific interference competition^45^. Finally, the Asian shore crab appears to enjoy enemy escape in its invaded range and is infected by fewer parasite species, and at lower prevalence, than conspecifics in its native range^46–48^. Thus, while all of these factors may be contributing to the recent declines in Asian shore crab populations, none of them stands out as a dominant driving force. We therefore examine the potential role of self-limitation via food reduction as an additional mechanism contributing to the Asian shore crab decline.

*H. sanguineus* has a generalist diet that includes primarily algae, mussels, barnacles, gastropods, and detritus^49–51^, and while their natural diet is often mostly herbivorous, they prefer to eat animal tissue when it is readily available^52^. Numerous studies have demonstrated the importance of diet across a wide range of brachyuran crab species for determining the extent of energy storage and reproductive success^17,53–57^. It therefore seems reasonable that energetics and reproduction in *H. sanguineus* should also be influenced by diet, and that this invader should generally be more successful when consumption rates are higher and/or when higher quality food is consumed. And indeed, energy storage in *H. sanguineus* increases strongly when consuming an animal diet compared to an algal diet^58^. However, the link between diet and reproductive success in *H. sanguineus* has not been clearly made, and there is a lack of correlation between energy storage and reproductive success in this species^59^. Additionally, the role of life history tradeoffs (e.g., between growth and reproduction) remains unclear for this invader. If a clear link can be made between the quality of diet and reproductive success, then this would suggest that self-limitation due to overexploitation of food resources is a contributing factor leading to the recent localized declines in *H. sanguineus* population sizes. Further, if food-induced life history tradeoffs can be demonstrated, then this would provide a plausible evolutionary mechanism in this species that could functionally induce self-limitation.

We examined long-term diet quality, energy storage, reproductive effort, and growth of individual *H. sanguineus* collected on the New Hampshire coast where this species has been present and abundant for 20 years. We sampled crabs across two sites that differ in community abundance and thus food availability in order to capture as wide a range of individual diet qualities as possible. For each crab, we assessed the general long-term dietary strategy and physiological growth and reproductive performance. We tested the general hypothesis that individual growth and reproduction would be greatest for individuals that consume a higher quality diet. We deconstruct this broad hypothesis into a series of more focused hypotheses, described below.

## Results

**Figure 1:**
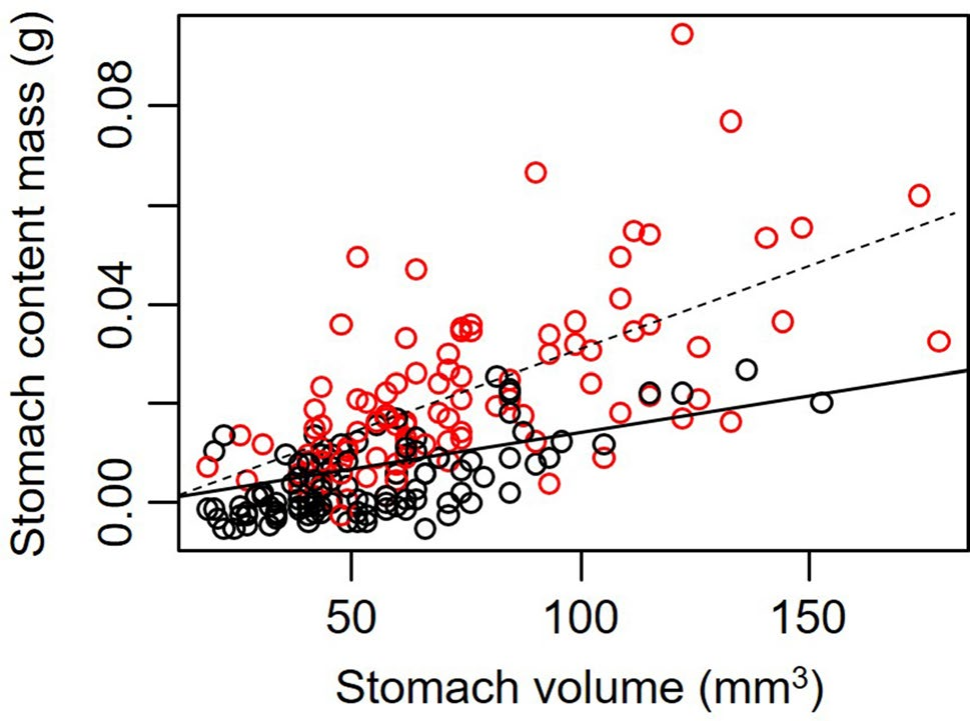
Mass of the stomach contents compared to calculated stomach volume for *Hemigrapsus sanguineus* captured at Odiorne Point (red circles, dashed line) and Ft. Stark (black circles, solid line).

**Figure 2:**
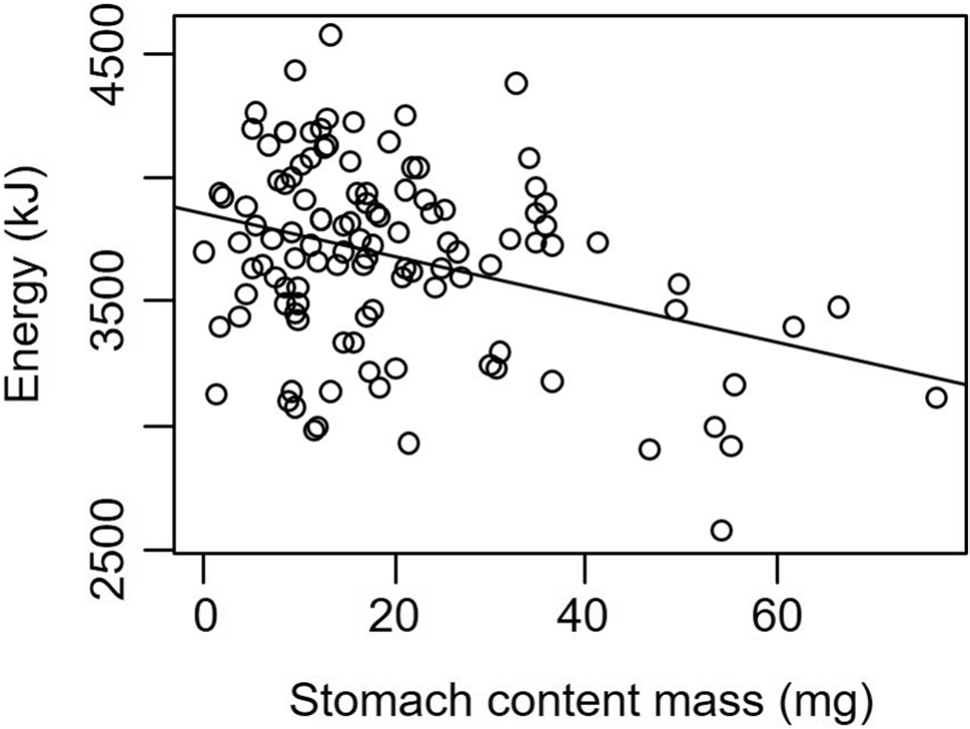
Energy content of food found in the stomach of *Hemigrapsus sanguineus* as a function of the amount of food consumed.

We found, consistent with our hypothesis, that crabs with larger stomach volumes consumed more food (*t* = 2.90, *P* = 0.004). However, while the main effect of site was not significant (*t* = − 0.43, *P* = 0.67), the interaction between CW and site was significant (*t* = 3.02, *P* = 0.003), indicating that the mass of food consumed increased faster with stomach volume for crabs at Odiorne Point than for crabs at Ft. Stark (Fig. 1). When each site was examined separately, we found that the mass of food in a crab’s stomach increased by 0.150 ± 0.027 mg and by 0.338 ± 0.042 mg for every 1-mm^3^ increase in stomach volume for crabs at Ft. Stark and at Odiorne Point, respectively (Fig. 1). However, we found that increasing the amount of food consumed did not translate into higher energy intake. Indeed, the energy content of food found in the stomach decreased by 8.58 ± 2.24 kJ with each additional mg of food consumed (*t* = − 3.83, *P* = 0.0002, Fig. 2).

**Figure 3:**
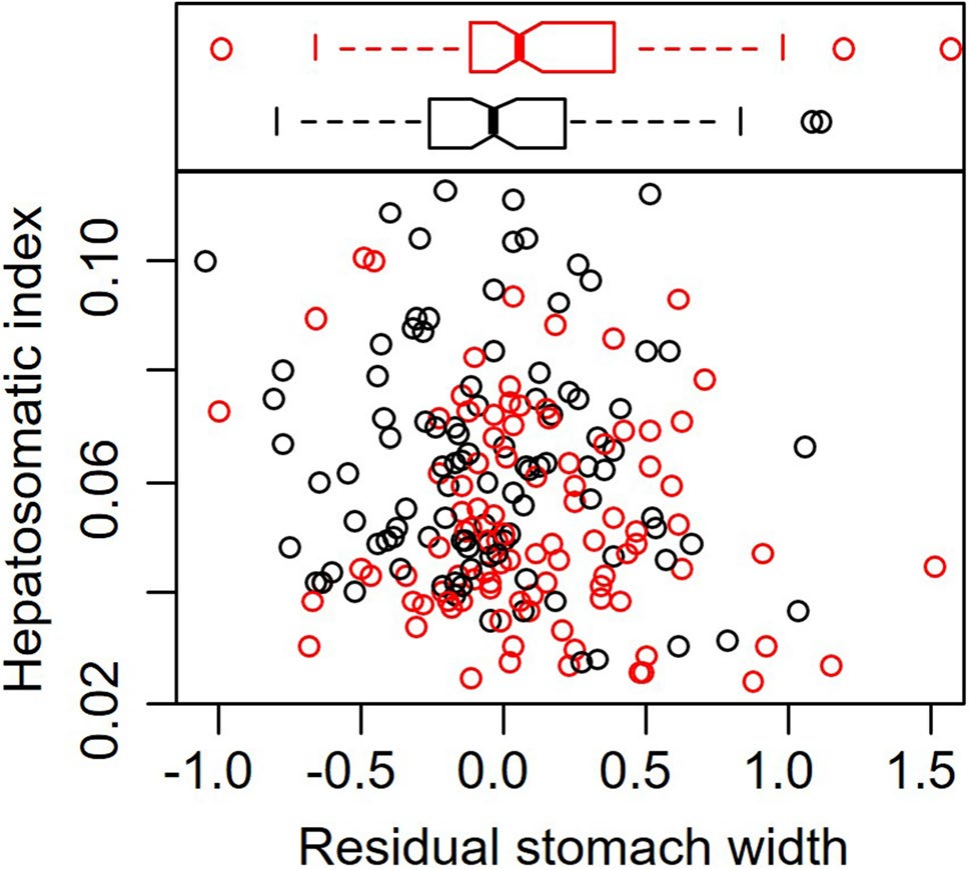
Relationship between the hepatosomatic index, or the proportion of total body weight comprised of the hepatopancreas, and residual stomach width after accounting for differences in stomach width with crab carapace width. Red circles indicate crabs from Odiorne Point; black circles indicate crabs from Ft. Stark. The horizontal boxplots at the top indicate residual SW for Odiorne Point (red) and Ft. Stark (black), where the solid line at the notch indicates the median value, the box encompasses the middle 50% of the data from the 1st to the 3rd quartile, the whiskers encompass 95% of the data, and the circles are outliers that fall outside this range.

As we explain in the Methods, we used the residual SW, after accounting for differences in SW with CW, as a proxy for long-term diet quality, where smaller residual SW indicates consumption of a consistently higher quality diet and larger residual SW indicates consumption of a consistently lower quality diet. On average, residual SW for crabs at Odiorne Point were larger than for crabs at Ft. Stark (*t* = 2.51, *P* = 0.006, Fig. 3). We found that crabs at Ft. Stark had higher mass-specific energy stores than crabs at Odiorne Point (*t* = − 3.61, *P* = 0.0004, Fig. 3). After accounting for differences across site, there was no additional impact of long-term diet quality on mass-specific energy storage (*t* = − 1.52, *P* = 0.13, Fig. 3).

**Figure 4:**
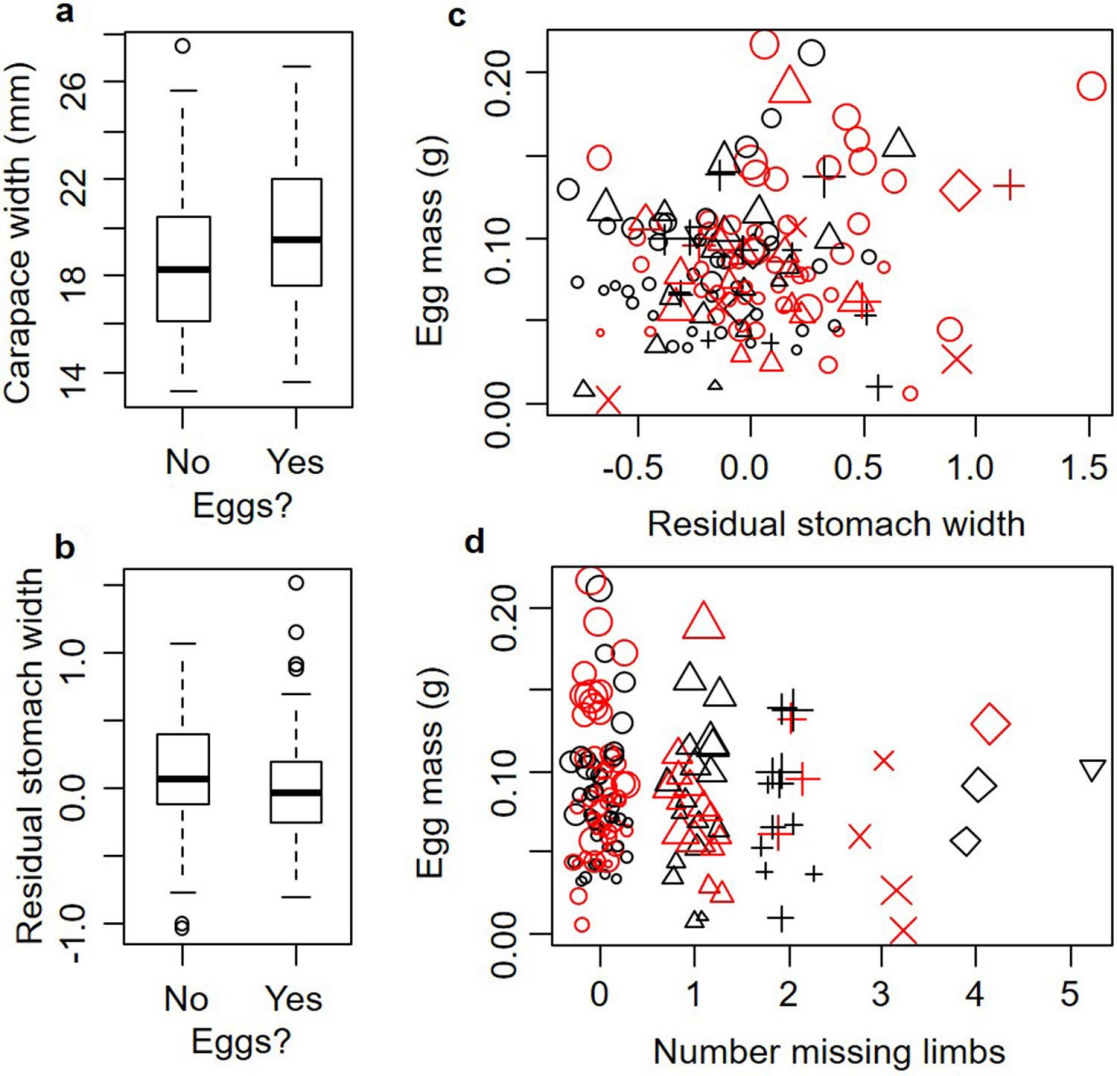
Influence of crab CW **(A)** and residual SW **(B)** on whether *Hemigrapsus sanguineus* are gravid. Boxplots interpreted as described in Fig. 3 caption. Relationship between the mass of the clutch of eggs and residual SW **(C)** and number of missing limbs **(D)**. In **(C,D)**: red and black symbols show Odiorne Point and Ft. Stark, respectively; symbol size shows relative crab CW, symbol type shows number of limbs missing (0: circle, 1: triangle, 2: plus sign, 3: X, 4: diamond, 5: inverted triangle). Symbols in **(D)** are jittered slightly in the x-direction to reduce overlap of data points for presentation purposes only.

Based on the generalized linear model with a binomial error distribution, we found that the odds of a crab being gravid increased as CW increased (*z* = 2.49, *P* = 0.013, Fig. 4a), decreased as the residual SW increased (*z* = − 3.00, *P* = 0.003, Fig. 4b), and was influenced by the interaction of these two predictor variables (*z* = 2.74, *P* = 0.006). We also found that the mass of the clutch of eggs was influenced by crab CW (*t* = 8.51, *P* < 0.0001), residual SW (*t* = − 2.59, *P* = 0.011), the number of limbs that were missing (*t* = 1.96, *P* = 0.053), the site of collection (*t* = 1.90, *P* = 0.059), as well as by several interactions between these variables. Specifically, crab CW interacted with residual SW (*t* = 2.74, *P* = 0.007), with the number of missing limbs (*t* = − 2.32, *P* = 0.022), and with collection site (*t* = − 2.31, *P* = 0.023). Further, residual SW interacted with the number of missing limbs (*t* = 1.99, *P* = 0.049). Lastly, three-way interactions were significant between CW, residual SW, and the number of missing limbs (*t* = − 2.21, *P* = 0.029) and between residual SW, the number of missing limbs, and site of collection (*t* = 2.82, *P* = 0.006, Fig. 4c). While these trends are complicated due to the number of interactions, inspection of Fig. 4c,d reveals that in general, clutch mass increased for larger crabs (larger symbols found towards the top of the Fig. 4c), increased with the quality of the diet (negative trend in clutch mass with residual SW in Fig. 4c), and decreased with the number of missing limbs (Fig. 4d).

**Figure 5:**
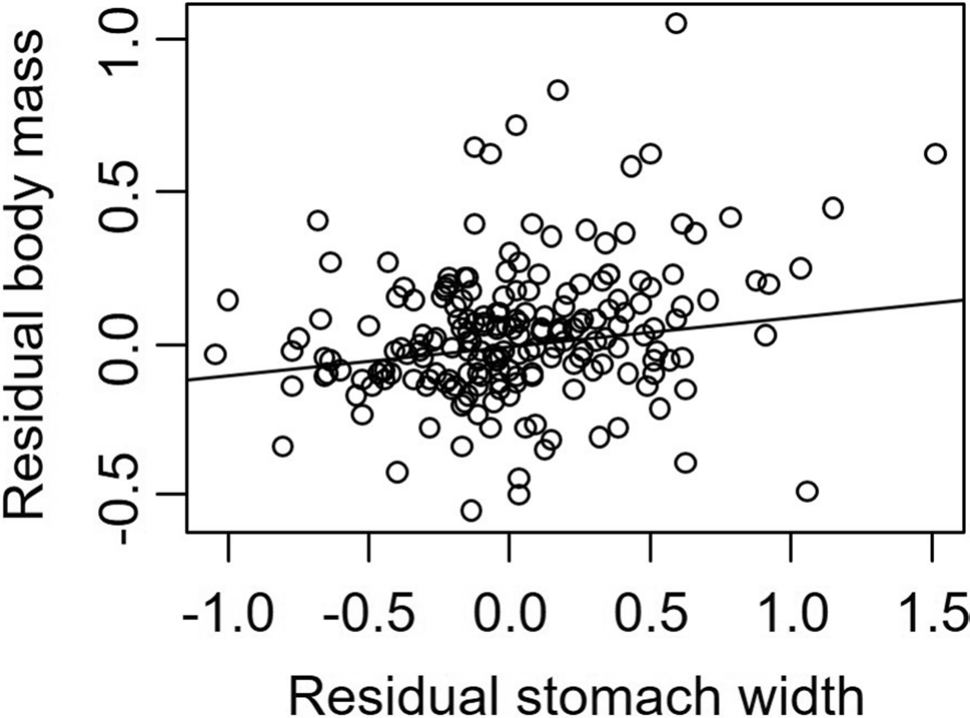
Residual total body mass (after accounting for changes with CW) as a function of residual SW for *Hemigrapsus sanguineus* for crabs collected at both sites.

Finally, we found that residual body mass (after accounting for CW) increased as the residual SW increased (*t* = 3.68, *P* = 0.003, Fig. 5). No other terms were included in the best-fitting model.

## Discussion

We have shown that the mass of consumed food increased with body size, but increased faster at Odiorne Point where high quality food is less available, and that there was an inverse correlation between the mass of food a crab consumed and the quality of that food. We have further demonstrated that body size and diet quality both influenced the likelihood that a crab was gravid, while the size of an egg clutch for gravid crabs increased with crab size, increased with higher diet quality (smaller residual SW), and decreased with injury, and these trends were generally seen across the two collection sites. Finally, we have shown that size-independent body mass increased as diet quality decreased, suggesting that individuals consuming a poorer quality diet have increased growth rates. These results are consistent with the concept of self-limitation in *H. sanguineus*, potentially driven by a tradeoff in energy allocation between reproduction and growth depending on diet quality.

While not in the context of invasive species, previous work has, however, examined self-limitation (often referred to as self-thinning) of sessile aquatic animals, such as mussels (e.g.^60^), barnacles^61^, and tunicates^62^. Mechanistically, such relationships can result from food competition at high densities^63^, analogous for these sessile animals to resource competition seen in plants that leads to a similar self-thinning phenomenon^11^. For these sessile plants and animals, scaling rules have been developed and debated that can be used to predict the relationship between density and biomass of individual organisms within a single age cohort^60,64^. Developing a similar scaling rule for mobile organisms would be challenging, as it would require density and biomass estimates across numerous replicate sites. Consequently, the concept of self-thinning or self-limitation for mobile organisms is not as thoroughly developed or demonstrated. And as highlighted in the Introduction, relatively few studies have successfully demonstrated self-limitation in mobile invasive species.

This is an observational study, and is thus open to alternative interpretations; however, our results are consistent with the concept of self-limitation in *H. sanguineus*. We have shown that reproduction tended to decrease in crabs that ate a lower quality diet, and in crabs that experienced higher levels of nonlethal injury (limb loss). Reduced diet quality and elevated nonlethal injury are both expected at high *H. sanguineus* densities due to depletion of food resources and increased conspecific aggression. These trends were apparent among individual crabs (the level of replication in this study), but also were consistent across the two sampling sites used here. Specifically, Odiorne Point has higher crab densities and lower per capita food availability, and crabs at this site generally consumed a lower quality diet and had lower reproductive rates (when comparing similarly sized crabs). Thus, reduced reproductive success, caused by depletion of high quality food, could help explain recent reductions in *H. sanguineus* population size in parts of its range where densities have previously been much higher.

Consumption by moderately high *H. sanguineus* densities (40 individuals m^−2^) can strongly reduce the entire algal and animal community^65^, and densities have historically been many times higher than this in areas where *H. sanguineus* has recently declined. At these high densities, *H. sanguineus* feeding activities greatly depress the prey community (e.g.^28,51,66^). If high densities of *H. sanguineus* depress prey abundances throughout a region, then the resulting low reproduction and regional recruitment could result in reduced population growth rates that could feasibly contribute to the population declines that have been documented.

Our results additionally suggest that self-limitation in *H. sanguineus* could be caused by a tradeoff in resource allocation that is driven by diet quality. Specifically, we found that there was a positive correlation between diet quality and reproductive success, but a negative correlation between diet quality and size-specific body mass (i.e., the amount of soft tissue growth since the last molt). This suggests that individuals that consume a higher quality diet allocate resources primarily to reproduction, while individuals that consume a lower quality diet may allocate resources primarily to soft tissue growth. This pattern is in direct opposition to the previously-suggested expectation that crustaceans should prioritize reproduction in resource-poor environments and growth in resource-rich environments^67^. Higher soft tissue mass for individuals with lower quality diet may also indicate postponement of molting, since diet quality is important for molting in crustaceans^68,69^. Consumption of a higher mass of lower quality food at Odiorne Point suggests that *H. sanguineus* is engaging in compensatory feeding to meet energy/nutrient demands at this site compared to at Ft. Stark. It is possible that this compensatory feeding triggers the tradeoff between resource allocation to growth vs. reproduction. Patterns consistent with this hypothesis have been shown in other systems, as dietary intake triggers a life history tradeoff in crickets, with increased dietary protein triggering allocation to reproduction, while increased dietary carbohydrates trigger allocation to muscle development to support migration^70^. Such strategies may evolve because diet composition determines the fatty acid composition of lipids that mothers deposit into eggs^17^, and fatty acid composition in turn influences development and survival of larvae^71^. Thus, when food quality is low, crabs may preferentially dedicate energy to growth rather than reproduction in order to increase size so that future reproductive attempts can be more prolific, given the allometric relationship between crab size and clutch size^72^.

The recent declines of *H. sanguineus* may have been influenced by factors other than self-limitation via food-depletion. For instance, mussel densities have declined in the Gulf of Maine over the last 40 years, possibly due to climate change^73^. The loss of this foundation species and the species it supports, would reduce food availability for *H. sanguineus* and other crabs inhabiting New England shores. Parasite infection may also play a role in limiting *H. sanguineus* success. This species is infected by both acanthocephalan and trematode parasites in its invaded range^48,74^, two groups of parasites that can impact the behavior and survival of crabs^75,76^. As highlighted in the Introduction, Asian shore crabs have fewer parasites in their invaded range than native species in the same area^47^ and fewer parasites than conspecifics in their native range^46–48^. However, while they have fewer parasites, they are more susceptible to infection^46^, and parasite load appears to be increasing since the initial escape from parasites at introduction, as native parasites in the invaded range expand to include this new host^47^. Similarly, while predation and competition have played a minor role in limiting the early invasion of this species^28,40,42–44^, it is possible that these processes may be taking on a greater role as time progresses, if native species learn to consume Asian shore crabs or more effectively interact with them. Thus, while our study does not suggest that resource limitation is the only, or even the predominant, mechanism leading to the decline of Asian shore crab populations, our results certainly do point to self-limitation because of food depletion as a contributing mechanism in the decline of this widespread invader.

## Methods

### Study sites

We collected crabs from Odiorne Point State Park in Rye, New Hampshire and from Fort Stark in New Castle, New Hampshire. These sites, located at the mouth of the Piscataqua River, are separated by only 2.3 km and are similar in many ways. Both are characterized by boulder fields overlying a mixed substrate of bedrock, cobble, and shell hash, and both support the same group of intertidal algal and animal species. However, Odiorne Point has more intertidal boulders than Ft. Stark, a habitat that is positively correlated with *H. sanguineus* abundance^49^. Consequently, *H. sanguineus* abundances averaged across all tidal heights at Odiorne Point (24.5 ± 18.8 m^−2^) are approximately 3X higher than those at Ft. Stark (7.5 ± 9.3 m^−2^)^77^. Further, while the two sites had nearly identical diversity and abundance of species within their intertidal communities prior to the arrival of *H. sanguineus*^78^, the higher density of *H. sanguineus* at Odiorne Point has had a greater impact on the prey community so that the abundance of each species is now lower at Odiorne Point than at Ft. Stark^65^ and references therein. The higher crab abundance and lower prey abundance at Odiorne Point means that the two sites differ considerably in the per capita amount of food available to *H. sanguineus*, particularly for animal prey, and therefore likely result in very different diets across the two sites. Our purpose for including crabs from both sites in our study was to capitalize on this difference in per capita prey availability in order to increase the range of diet strategies observed across crabs as much as possible. In all statistical analyses, we included site as a categorical independent variable to explicitly account for the fact that crabs were sampled across these two different sites.

### Female performance

We sampled adult female crabs haphazardly from each site (n = 99 from Odiorne Point, n = 102 from Fort Stark). *H. sanguineus* forages most actively at night^79^, and so we sampled at dawn on receding tides to maximize the likelihood that there would be recently-consumed food in the stomach. We collected crabs by hand by turning over boulders to collect from intertidal regions of the shore. Upon collection, crabs were frozen in individual Ziploc bags and were returned on dry ice to Brigham Young University in Provo, UT for further analysis. *H. sanguineus* reaches maturity at 12.1 mm carapace width^80^, and only crabs exceeding this size were collected. Prior to dissection, we measured the carapace width (CW) of each crab to the nearest 0.1 mm and noted any missing or regenerating limbs. We then dissected crabs by first removing the dorsal carapace. Then we separated the hepatopancreas, cardiac stomach, egg clutch if the female had any, the ovaries, and the remainder of the body into separate pre-weighed tins that were dried to a constant weight at 65 °C and were then weighed to the nearest 0.01 mg. Prior to drying, we also measured the cardiac stomach width (SW) on the dorsal, anterior margin to the nearest 0.1 mm. All statistical tests described below were conducted using R v. 3.6.0^81^.

We first analyzed the cardiac stomach to determine a snapshot of the amount of food consumed and how this was related to crab stomach size and collection site. We determined the mass of food consumed, independent of the mass of the stomach wall itself, as follows. We categorized the stomach as empty or not, based on visual observation through the transparent stomach wall. Using the subset of stomachs that were empty (n = 11), we determined the linear equation relating mass of the empty stomach to crab size (stomach mass = 0.0018 × CW – 0.0123, R^2^ = 0.71, *P* = 0.001) and used this to determine the mass of the stomach wall for each crab. For crabs with food in their stomachs, we then determined the mass of food consumed as the stomach total dry mass, minus the mass of the stomach wall using the relationship given above. This value inherently has error when used as a proxy of amount consumed due to variable digestibility of consumed food, time since consumption across crabs, and temporal variation in consumption amount from day to day; however, it provides a rough estimate of the relative amount of food consumed by individual crabs. We converted SW to stomach volume using the following relationship empirically determined by Griffen and Mosblack^82^:

$$\text{Stomach volume}=0.92\frac{\sqrt{2}}{12}{SW}^{3}$$

We tested the hypothesis that crabs with larger stomachs would eat more using a linear model, with stomach content mass as response variable, and using stomach volume and site, and their interaction, as predictor variables, and only using crabs where stomach content mass > 0.

The mass of the stomach content indicates how much food was consumed, but says nothing about diet quality. We used energy content as a proxy for diet quality and determined the quality of the most recent meal by measuring the energy content of food found in the cardiac stomach. We combusted each stomach that contained food in a Parr 6725 semi-micro oxygen bomb calorimeter, yielding the energy content of each stomach. We ignored the energy content of the stomach wall itself because the mass of the empty stomach was below the detection limit of the calorimeter and therefore could not be combusted. The mass of food in some crab stomachs was very small, also falling below the detection limit of the calorimeter. For these, we paired nearly-empty stomachs from crabs collected at the same site and combusted the pairs together. We used a linear model to test the hypothesis that energy content (kJ) increased with the mass of food in the stomach, again only using crabs where stomach content mass > 0. We initially included sampling site in the analysis, but it was not significant (*P* > 0.4) and so it was removed.

The energetic content of the stomach mass just described provides an estimate of the diet quality of the last meal, but it is likely that the diet of an individual crab varies through time. The width of the cardiac stomach in crabs, and thus the stomach volume via the relationship given in the equation above, is strongly related to long-term diet. Specifically, Griffen and Mosblack^82^ demonstrated across 15 crab species that stomach size decreased with the amount of animal tissue included in the diet. This is likely because herbivorous crabs are generally nitrogen-limited^83^, and plants are often much lower in nitrogen than animal tissue^84^. Thus, crabs with a lower quality diet must consume a greater quantity to meet their nutritional needs (i.e., compensatory feeding). These differences in stomach size are evident throughout ontogeny, as crab species adapted to a more herbivorous diet start life with larger cardiac stomachs and experience faster allometric or isometric growth of the cardiac stomach throughout ontogeny compared to more carnivorous species^85^. Further, these diet-related patterns in stomach size between species are mirrored by differences between individuals within a single species. In a field experiment with *H. sanguineus*, Griffen and Mosblack^82^ showed that substantial diet variation exists between individual crabs, and that individual crabs that preferentially consumed animal material rather than algae, had smaller cardiac stomachs. Thus, intraspecific differences in long-term diet trends in this and other crab species^17,86^ can reliably be quantified by comparing differences in the width of the cardiac stomach, after controlling for differences in crab body size.

We assessed long-term diet quality of individual crabs using the width of the cardiac stomach. Stomach width increases linearly with CW in *H. sanguineus*^85^, but there is variation around the mean relationship. We therefore used this variation (i.e., the residual SW after accounting for CW) as a proxy for diet quality of individual crabs in the analyses that follow. We compared the general diet quality for crabs at the two sites by comparing residual SW using a t-test. We used a one-sided t-test because we expected the diet quality to be higher at Ft. Stark because, as described above, the per capita availability of animal prey is higher there^77^.

We used the mass of the hepatopancreas, an energy storage organ in crabs^87^, to test the hypothesis that energy storage differed with diet. We calculated the hepatosomatic index as the mass of the hepatopancreas divided by the mass of the crab^88^. We then used this index as the response variable in a linear model with residual SW and sampling site treated as predictor variables. We initially included the interaction term, but it did not explain a significant amount of the variation and so was removed.

We tested the hypothesis that reproduction varies with diet quality using the mass of eggs as our response variable. We chose to analyze eggs rather than ovaries because we reasoned that variation in ovary mass would be partially explained by time since the last egg clutch had been produced, which was unknowable for crabs not carrying a clutch of eggs when they were collected. There were a large number of crabs that were not gravid, resulting in a zero-inflated dataset. We therefore used a “hurdle” or “two-stage” modeling approach^89^. Specifically, we first used a generalized linear model with a binomial distribution (i.e., a logistic regression) to determine whether a crab was gravid (yes/no). Second, we used a linear model with the mass of the egg clutch as the response, including only those crabs that had an egg clutch. We included the following predictor variables and took the following approach in both of these analyses. We included CW because clutch size is expected to increase with crab size. We included the number of missing limbs because we expected that crabs that were regenerating limbs would have less energy to devote to reproduction. We included residual SW to examine our hypothesis that long-term diet strategy influences reproduction. And finally, we included site. We also included all possible interactions to create a full model and then compared the full model and all possible simpler models using AIC. In each case, we selected the best fitting model as the one with the lowest AIC and with ΔAIC for all other models being > 2.

Finally, we also examined the influence of diet quality on body mass as a proxy for growth. Crabs grow incrementally by periodically shedding their exoskeleton and growing a larger one, but in between molts, individuals grow soft tissue continually^90^. Thus, we examined the total body mass as the mass of the dry body, hepatopancreas, ovaries, eggs, and missing legs (i.e., all body parts with the exclusion of the full cardiac stomach so as not to include the mass of consumed food). We included the mass of missing legs to ensure that any differences between crabs were driven by differences in soft tissue mass and not differences in the number of missing legs. Thus, adding back in the mass of the missing legs reduces differences in mass between crabs and provides a conservative comparison of growth differences. We determined the mass of the missing legs by individually weighing each of the eight walking legs and the two claws from each of 30 different crabs of a range of CW. We then determined the regression equation for the mass of each of these 10 limbs as a function of CW (range of R^2^ values from the 10 regressions: 0.845–0.942). We then determined the mass of any missing leg for each crab using the appropriate regression equation for that specific leg. Total body mass increased allometrically with CW according to the equation: total body mass = 0.0027 × CW^2.093^. We therefore determined residual total body mass for each crab, after accounting for CW using this equation. Crabs with a larger residual body mass would have experienced more growth since the last molt, and we therefore used this residual body mass as the response variable in a linear model with residual SW, site, and their interaction as predictor variables; however, the interaction term and the main effect of site were not significant, and so were sequentially removed.

## References

[1] Bowman, W.D., Hacker, S.D., Cain, M.L. *Ecology*, 4th Edn. (Sinauer Press, 2017).

[2] Eggleston DB, Lipcius RN, Hines AH (1992). Density-dependent predation by blue crabs upon infaunal clam species with contrasting distribution and abundance patterns. Mar. Ecol. Progr. Ser..

[3] Boström-Einarsson L, Bonin MC, Munday PL, Jones GP (2013). Strong intraspecific competition and habitat selectivity influence abundance of a coral-dwelling damselfish. J. Exp. Mar. Biol. Ecol..

[4] Ruggerone, G.T., Zimmermann, M., Myers, K.W., Nielsen, J.L., & Rogers, D.E. Competition between Asian pink salmon (*Oncorhynchus gorbuscha*) and Alaskan sockeye salmon (*O. nerka*) in the North Pacific Ocean. *Fish. Oceanogr.* **12**, 209–219 (2003).

[5] Greer AL, Briggs CJ, Collins JP (2008). Testing a key assumption of host-pathogen theory: Density and disease transmission. Oikos.

[6] Turchin P (2001). Does population ecology have general laws?. Oikos.

[7] Yenni G, Adler PB, Ernest SM (2012). Strong self-limitation promotes the persistence of rare species. Ecology.

[8] Weis AE, Simms EL, Hochberg ME (2000). Will plant vigor and tolerance be genetically correlated? Effects of intrinsic growth rate and self-limitation on regrowth. Evol. Ecol..

[9] Marino A, Rodríguez V, Pazos G (2016). Resource-defense polygyny and self-limitation of population density in free-ranging guanacos. Behav. Ecol..

[10] Chamaillé-Jammes S, Fritz H, Valeix M, Murindagomo F, Clobert J (2008). Resource variability, aggregation and direct density dependence in an open context: The local regulation of an African elephant population. J. Anim. Ecol..

[11] Westoby M (1984). The self-thinning rule. Adv. Ecol. Res..

[12] Sedinger, J. S., Herzog, M. P., Person, B. T., Kirk, M. T., Obritchkewitch, T., Martin, P. P., & Bosque, C. Large-scale variation in growth of Black Brant goslings related to food availability. *Auk* **118**, 1088–1095 (2001).

[13] Marschall EA, Crowder LB (1995). Density-dependent survival as a function of size in juvenile salmonids in streams. Can. J. Fish. Aquat. Sci..

[14] Zheng X, Huang L, Huang B, Lin Y (2013). Factors regulating population dynamics of the amphipod *Ampithoe valida* in a eutrophic subtropical coastal lagoon. Acta Oceanol. Sin..

[15] Li, G. Y., & Zhang, Z. Q. Does size matter? Fecundity and longevity of spider mites (*Tetranychus urticae*) in relation to mating and food availability. *Syst. Appl. Acarol.-UK* **23**, 1796–1808 (2018).

[16] Niu H, Zhao L, Sun J (2013). Phenotypic plasticity of reproductive traits in response to food availability in invasive and native species of nematode. Biol. Inv..

[17] Cannizzo ZJ, Lang SQ, Benitez-Nelson B, Griffen BD (2020). An artificial habitat increases the reproductive fitness of a range-shifting species within a newly colonized ecosystem. Sci. Rep..

[18] Zera AJ, Harshman LG (2001). The physiology of life history trade-offs in animals. Annu. Rev. Ecol. Evol. Syst..

[19] Strayer, D. L., D'Antonio, C. M., Essl, F., Fowler, M. S., Geist, J., Hilt, S., & Latzka, A. W. Boom‐bust dynamics in biological invasions: Towards an improved application of the concept. *Ecol. Lett.* **20**, 1337–1350 (2017). 10.1111/ele.12822 28834087

[20] Jaćimović M, Lenhardt M, Krpo-Ćetković J, Jarić I, Gačić Z, Hegediš A (2019). Boom-bust like dynamics of invasive black bullhead (*Ameiurus melas*) in Lake Sava (Serbia). Fish. Manag. Ecol..

[21] Alcorlo, P., Geiger, W., & Otero, M. Reproductive biology and life cycle of the invasive crayfish *Procambarus clarkii* (Crustacea: Decapoda) in diverse aquatic habitats of South-Western Spain: Implications for population control. *Fund. Appl. Limnol./Arch. Hydrobiol.* **173**, 197–212 (2008).

[22] Melero, Y., Robinson, E., & Lambin, X. Density-and age-dependent reproduction partially compensates culling efforts of invasive non-native American mink. *Biol. Invasions* **17**, 2645–2657.

[23] Yoshida K, Hoshikawa K, Wada T, Yusa Y (2013). Patterns of density dependence in growth, reproduction and survival in the invasive freshwater snail *Pomacea canaliculata* in Japanese rice fields. Freshw. Biol..

[24] Williams AB, McDermott JJ (1990). An eastern United States record for the western Indo-Pacific crab, *Hemigrapsus sanguineus* (Crustacea: Decapoda: Grapsidae). Proc. Biol. Soc. Wash..

[25] Breton G, Faasse M, Noël P, Vincent T (2002). A new alien crab in Europe: *Hemigrapsus sanguineus* (Decapoda: Brachyura: Grapsidae). J. Crustacean Biol..

[26] Blakeslee, A. M., Kamakura, Y., Onufrey, J., Makino, W., Urabe, J., Park, S., & Miura, O. Reconstructing the invasion history of the Asian shorecrab, *Hemigrapsus sanguineus* (De Haan 1835) in the Western Atlantic. *Mar. Biol.* ***164***, 47 (2017).

[27] Lohrer AM, Whitlatch RB (2002). Interactions among aliens: Apparent replacement of one exotic species by another. Ecology.

[28] Kraemer GP, Sellberg M, Gordon A, Main J (2007). Eight-year record of *Hemigrapsus sanguineus* (Asian shore crab) invasion in western Long Island Sound estuary. Northeast. Nat..

[29] Epifanio CE (2013). Invasion biology of the Asian shore crab *Hemigrapsus sanguineus*: A review. J. Exp. Mar. Biol. Ecol..

[30] Lord JP, Williams LM (2017). Increase in density of genetically diverse invasive Asian shore crab (*Hemigrapsus sanguineus*) populations in the Gulf of Maine. Biol. Invasions.

[31] Brousseau DJ, Kriksciun K, Baglivo JA (2003). Fiddler crab burrow usage by the Asian crab, *Hemigrapsus sanguineus*, in a Long Island Sound salt marsh. Northeast. Nat..

[32] O’Connor NJ (2014). Invasion dynamics on a temperate rocky shore: from early invasion to establishment of a marine invader. Biol. Invasions.

[33] O'Connor NJ (2018). Changes in population sizes of *Hemigrapsus sanguineus* (Asian Shore Crab) and resident crab species in southeastern New England (2010–2016). Northeast. Nat..

[34] Schab CM, Park S, Waidner LA, Epifanio CE (2013). Return of the native: Historical comparison of invasive and indigenous crab populations near the mouth of Delaware Bay. J. Shellfish Res..

[35] Bloch CP, Curry KD, Fisher-Reid MC, Surasinghe TD (2019). Population Decline of the Invasive Asian Shore Crab (*Hemigrapsus sanguineus*) and Dynamics of Associated Intertidal Invertebrates on Cape Cod, Massachusetts. Northeast. Nat..

[36] Kraemer GP (2019). Changes in population demography and reproductive output of the invasive *Hemigrapsus sanguineus* (Asian Shore Crab) in the Long Island Sound from 2005 to 2017. Northeast. Nat..

[37] Stentiford, G. D., Bateman, K. S., Dubuffet, A., Chambers, E., & Stone, D. M. *Hepatospora eriocheir* (Wang and Chen, 2007) gen. et comb. nov. infecting invasive Chinese mitten crabs (*Eriocheir sinensis*) in Europe. *J. Invertebr. Pathol.* **108**, 156–166 (2011). 10.1016/j.jip.2011.07.008 21854783

[38] Bateman AW, Buttenschön A, Erickson KD, Marculis NG (2017). Barnacles vs bullies: Modelling biocontrol of the invasive European green crab using a castrating barnacle parasite. Theor. Ecol..

[39] Bojko, J., Stebbing, P. D., Dunn, A. M., Bateman, K. S., Clark, F., Kerr, Stewart-Clark, S., Johannesen, Á., & Stentiford, G. D. Green crab *Carcinus maenas* symbiont profiles along a North Atlantic invasion route. *Dis. Aquat. Organ.* **128**, 147–168 (2018). 10.3354/dao03216 29733028

[40] Jensen GC, McDonald PS, Armstrong DA (2002). East meets west: competitive interactions between green crab *Carcinus maenas*, and native and introduced shore crab *Hemigrapsus* spp. Mar. Ecol. Progr. Ser..

[41] DeRivera CE, Ruiz GM, Hines AH, Jivoff P (2005). Biotic resistance to invasion: Native predator limits abundance and distribution of an introduced crab. Ecology.

[42] Kim AK, O'Connor NJ (2007). Early stages of the Asian shore crab *Hemigrapsus sanguineus* as potential prey for the striped killifish *Fundulus majalis*. J. Exp. Mar. Biol. Ecol..

[43] Brousseau DJ, Murphy AE, Enriquez NP, Gibbons K (2008). Foraging by two estuarine fishes, *Fundulus heteroclitus* and *Fundulus majalis*, on juvenile Asian shore crabs (*Hemigrapsus sanguineus*) in Western Long Island Sound. Estuar. Coast..

[44] Savaria MC, O'Connor NJ (2013). Predation of the non-native Asian shore crab *Hemigrapsus sanguineus* by a native fish species, the cunner (*Tautogolabrus adspersus*). J. Exp. Mar. Biol. Ecol..

[45] Griffen BD, Delaney DG (2007). Species invasion shifts the importance of predator dependence. Ecology.

[46] Keogh CL, Miura O, Nishimura T, Byers JE (2017). The double edge to parasite escape: invasive host is less infected but more infectable. Ecology.

[47] Kroft KL, Blakeslee AM (2016). Comparison of parasite diversity in native panopeid mud crabs and the invasive Asian shore crab in estuaries of northeast North America. Aquat. Invasions.

[48] Blakeslee AM, Keogh CL, Byers JE, Lafferty AMKKD, Torchin ME (2009). Differential escape from parasites by two competing introduced crabs. Mar. Ecol. Progr. Ser..

[49] Lohrer AM, Fukui Y, Wada K, Whitlatch RB (2000). Structural complexity and vertical zonation of intertidal crabs, with focus on habitat requirements of the invasive Asian shore crab, *Hemigrapsus sanguineus* (de Haan). J. Exp. Mar. Biol. Ecol..

[50] Ledesma ME, O'Connor NJ (2001). Habitat and diet of the non-native crab *Hemigrapsus sanguineus* in southeastern New England. Northeast. Nat..

[51] Brousseau DJ, Goldberg R (2007). Effect of predation by the invasive crab *Hemigrapsus sanguineus* on recruiting barnacles *Semibalanus balanoides* in western Long Island Sound, USA. Mar. Ecol. Progr. Ser..

[52] Brousseau DJ, Baglivo JA (2005). Laboratory investigations of food selection by the Asian shore crab, *Hemigrapsus sanguineus*: Algal versus animal preference. J. Crustacean Biol..

[53] Griffen BD (2014). Linking individual diet variation and fecundity in an omnivorous marine consumer. Oecologia.

[54] Riley ME, Vogel M, Griffen BD (2014). Fitness-associated consequences of an omnivorous diet for the mangrove tree crab *Aratus pisonii*. Aquat. Biol..

[55] Griffen BD, Norelli AP (2015). Spatially variable habitat quality contributes to within-population variation in reproductive success. Ecol. Evol..

[56] Griffen BD, Riley ME (2015). Potential impacts of invasive crabs on one life history strategy of native rock crabs in the Gulf of Maine. Biol. Invasions.

[57] Belgrad BA, Griffen BD (2016). The influence of diet composition on fitness of the blue crab, *Callinectes sapidus*. PLoS ONE.

[58] Griffen BD, Vogel M, Goulding L, Hartman R (2015). Energetic effects of diet choice by invasive Asian shore crabs: Implications for persistence when prey are scarce. Mar. Ecol. Progr. Ser..

[59] Griffen BD (2018). The timing of energy allocation to reproduction in an important group of marine consumers. PLoS ONE.

[60] Guiñez R, Petraitis PS, Castilla JC (2005). Layering, the effective density of mussels and mass-boundary curves. Oikos.

[61] Bertness MD, Gaines SD, Yeh SM (1998). Making mountains out of barnacles: The dynamics of acorn barnacle hummocking. Ecology.

[62] Guiñez R, Castilla JC (2001). An allometric tridimensional model of self-thinning for a gregarious tunicate. Ecology.

[63] Alunno-Bruscia M, Petraitis PS, Bourget E, Fréchette M (2000). Body size–density relationship for *Mytilus edulis* in an experimental food-regulated situation. Oikos.

[64] Weller, D. E. A reevaluation of the‐3/2 power rule of plant self‐thinning. *Ecol. Monogr.* **57**, 23–43 (1987).

[65] Griffen BD, Byers JE (2009). Community impacts of two invasive crabs: the interactive roles of density, prey recruitment, and indirect effects. Biol. Invasions.

[66] Lohrer AM, Whitlatch RB (2002). Relative impacts of two exotic brachyuran species on blue mussel populations in Long Island Sound. Mar. Ecol. Progr. Ser..

[67] Nelson K (1991). Scheduling of reproduction in relation to molting and growth in malacostracan crustaceans. Crustacean Egg Product..

[68] Kibria G (1993). Studies on molting, molting frequency and growth of shrimp *Penaeus monodon* fed on natural and compounded diets. Asian Fish. Sci..

[69] Petit H, Nègre-Sadargues G, Castillo R, Trilles JP (1997). The effects of dietary astaxanthin on growth and moulting cycle of postlarval stages of the prawn, *Penaeus japonicus* (Crustacea, Decapoda). Comp. Biochem. Physiol. A Physiol..

[70] Clark RM, Zera AJ, Behmer ST (2015). Nutritional physiology of life-history trade-offs: How food protein–carbohydrate content influences life-history traits in the wing-polymorphic cricket *Gryllus firmus*. J. Exp. Biol..

[71] Rosa R, Calado R, Narciso L, Nunes ML (2007). Embryogenesis of decapod crustaceans with different life history traits, feeding ecologies and habitats: A fatty acid approach. Mar. Biol..

[72] Hines AH (1982). Allometric constraints and variables of reproductive effort in brachyuran crabs. Mar. Biol..

[73] Sorte, C. J., Davidson, V. E., Franklin, M. C., Benes, K. M., Doellman, M. M., Etter, R. J., & Menge, B. A. Long‐term declines in an intertidal foundation species parallel shifts in community composition. *Global Change Biol.* **23**, 341–352 (2017). 10.1111/gcb.13425 27411169

[74] Goedknegt, M. A., Havermans, J., Waser, A. M., Luttikhuizen, P. C., Velilla, E., Camphuysen, K. C., & Thieltges, D. W. Cross-species comparison of parasite richness, prevalence, and intensity in a native compared to two invasive brachyuran crabs. *Aquat. Invasions* **12**, 201–212 (2017).

[75] Latham A, Poulin R (2002). Field evidence of the impact of two acanthocephalan parasites on the mortality of three species of New Zealand shore crabs (Brachyura). Mar. Biol..

[76] Latham ADM, Poulin R (2002). Effect of acanthocephalan parasites on hiding behaviour in two species of shore crabs. J. Helminthol..

[77] Griffen, B. D., van den Akker, D., NiNuzzo, E. R., Anderson, L. III, & Vernier, A. Comparing methods for predicting the impacts of invasive species **(in press)**.

[78] Tyrrell, M.C., & Harris, L.G. Potential impact of the introduced Asian shore crab, *Hemigrapsus sanguineus*, in northern New England: Diet, feeding preferences, and overlap with the green crab, *Carcinus maenas*. in *Marine Bioinvasions: Proceedings of the First National Conference, Cambridge, MA, 24–27 January 1999* (pp. 208–220). (MIT Sea Grant College Program, 2000).

[79] Spilmont N, Gothland M, Seuront L (2015). Exogenous control of the feeding activity in the invasive Asian shore crab *Hemigrapsus sanguineus* (De Haan, 1835). Aquat. Invasions.

[80] McDermott JJ (1998). The western Pacific brachyuran *Hemigrapsus sanguineus* (Grapsidae) in its new habitat along the Atlantic coast of the United States: reproduction. J. Crustacean Biol..

[81] R Core Team. *R: A Language and Environment for Statistical Computing*. (R Foundation for Statistical Computing, Vienna, 2019). https://www.R-project.org/.

[82] Griffen BD, Mosblack H (2011). Predicting diet and consumption rate differences between and within species using gut ecomorphology. J. Anim. Ecol..

[83] Wolcott DL, O'Connor NJ (1992). Herbivory in crabs: Adaptations and ecological considerations. Am. Zool..

[84] Mattson WJ (1980). Herbivory in relation to plant nitrogen content. Annu. Rev. Ecol. Evol. Syst..

[85] Griffen BD, Cannizzo ZJ, Gül MR (2018). Ecological and evolutionary implications of allometric growth in stomach size of brachyuran crabs. PLoS ONE.

[86] Gül MR, Griffen BD (2020). Diet, energy storage, and reproductive condition in a bioindicator species across beaches with different levels of human disturbance. Ecol. Indic..

[87] Vogt G (2019). Functional cytology of the hepatopancreas of decapod crustaceans. J. Morphol..

[88] Kyomo J (1988). Analysis of the relationship between gonads and hepatopancreas in males and females of the crab *Sesarma intermedia*, with reference to resource use and reproduction. Mar. Biol..

[89] Zuur, A. F., Ieno, E. N., Walker, N. J., Saveliev, A. A., Smith, G. M. Zero-truncated and zero-inflated models for count data. in *Mixed Effects Models and Extensions in Ecology with R* 261–293. (Springer, New York, 2009).

[90] Mente E (2003). Effect of ration level on individual food consumption, growth and protein synthesis in the shore crab *Carcinus maenas*. Nutrition, Physiology and Metabolism of Crustaceans.

